# Journal of Health Monitoring – what’s new in 2024?

**DOI:** 10.25646/12201

**Published:** 2024-06-26

**Authors:** Thomas Ziese, Anke-Christine Saß

**Affiliations:** Robert Koch Institute, Department of Epidemiology and Health Monitoring, Berlin, Germany

**Dear readers,**

In 2016, the Journal of Health Monitoring was launched – a scientific journal for public health topics, aimed in particular at stakeholders in health care, politics and science. All articles are open access. They are available online in English and German. The Journal is published by the Robert Koch Institute (RKI) as part of the Federal Health Reporting in Germany (Gesundheitsberichterstattung, GBE).

Until then, the GBE had mainly published printed reports. The time was ripe for a new, digital format. A landscape layout was developed, customised for reading on a screen, with interactive features such as links to tables on the internet. The first issue in September 2016 included four articles on alcohol use by adults – still a current topic.

To date, around 300 articles have been published in the Journal. In addition to original research, such as data-based analyses and reviews, the Journal publishes methodological articles, conference papers and guidelines. Each issue focuses on a key topic of high public health relevance. In the current issue, it is diabetes mellitus, a research focus of the RKI and an important component of the National Public Health Surveillance for Non-Communicable Diseases and their influencing factors, which is currently being established at the Institute.

Over the past years, our editorial team has continuously worked on the processes and standards and on the development of the Journal. We are guided in particular by the Recommendations for the Conduct, Reporting, Editing, and Publication of Scholarly Work in Medical Journals of the International Committee of Medical Journal Editors (ICMJE) [1] and the Principles of Transparency and Best Practice in Scholarly Publishing [2].

This year, the changes are particularly visible: the layout of the Journal has been changed to a portrait format. Your feedback as readers has contributed to this decision. The most important information about the Journal and articles now appear on the first page, such as the license terms and peer review status. Accessibility requirements have played an important role in the redesign. Also new is the Journal’s publication schedule, which has changed from issue-based to continuous publication. This will allow scientific knowledge to be disseminated more quickly, a major advantage of the online format. All articles are now published as soon as they are ready. Published articles roll up into four issues per year, featuring several articles on a key topic.

The international visibility of the articles continues to be an important concern for us. The Journal is therefore archived in PubMed Central (J Health Monit) [3] and the English articles can be found via the PubMed search. We are particularly pleased that the indexing of the English articles in the Directory of Open Access Journals (DOAJ) [4] has been accompanied by the award of the DOAJ seal for best practice in open access publishing – a recognition that only about ten percent of the journals indexed in the DOAJ receive [5].

We would like to take this opportunity to thank all those who contribute to the success of the Journal of Health Monitoring. We want to thank our authors for their contributions and for their willingness to keep our GBE readers in mind by presenting information in a way that is readily comprehensible – a balancing act that is not always easy in scientific publications. We would also like to thank the reviewers who offer their time and expertise to provide an independent and critical assessment of the manuscripts in a double-anonymous peer review process – an important part of the scientific process. We are grateful to the members of the Editorial Board, who support us in the editorial direction of the Journal and provide important input for topic planning. And last but not least, a special thank you goes to the dedicated editorial, coordination, layout and accessibility team that has been realising the Journal for eight years. The great commitment and creativity of these colleagues has made it possible for the Journal to be well established today. Our work associated with the Journal continues to be a great pleasure.

Finally, a brief outlook: The Journal is an important part of the Federal Health Reporting. A single format can, however, only cover part of the addressees and needs. A central web platform is currently being set up at the RKI to provide access to comprehensive information on non-communicable diseases and their determinants. Among other things, the website will present an interactive visualisation of public health surveillance indicators for non-communicable diseases. Structured access to publications, reports and other formats will also be established, so that the Journal will also be available via this portal in the future.

We look forward to continuing our exchange with you at future conferences and workshops and to hearing your wishes and suggestions for the Journal of Health Monitoring. You are also welcome to write to us at healthmonitoring@rki.de. And please subscribe to our newsletter at www.rki.de/gbe-newsletter-en if you want to be informed about new articles in the Journal and other news from the GBE.
